# Lessons learned from stakeholders in a facilitation intervention targeting neonatal health in Quang Ninh province, Vietnam

**DOI:** 10.1186/1471-2393-13-234

**Published:** 2013-12-13

**Authors:** Leif Eriksson, Duong M Duc, Ann Catrine Eldh, Vu Pham N Thanh, Tran Q Huy, Mats Målqvist, Lars Wallin

**Affiliations:** 1 Department of Women’s and Children’s Health, International Maternal and Child Health (IMCH), Uppsala University, SE-751 85, Uppsala, Sweden; 2 Hanoi School of Public Health, 138 Giang Vo, Ba Dinh, Hanoi, Vietnam; 3 Department of Neurobiology, Care Sciences and Society, Division of Nursing, Karolinska Institutet, SE-171 77, Stockholm, Sweden; 4 Public Health & Environment Department, Institute of Sociology, 01 Lieu Giai, Ba Dinh, Hanoi, Vietnam; 5 Department of Medical Services Administration, Ministry of Health, Nursing office, 138A Giang Vo, Ba Dinh, Hanoi, Vietnam; 6 School of Health and Social Studies, Dalarna University, SE-791 88, Falun, Sweden

## Abstract

**Background:**

In northern Vietnam the Neonatal health - Knowledge Into Practice (NeoKIP, Current Controlled Trials ISRCTN44599712) trial has evaluated facilitation as a knowledge translation intervention to improve neonatal survival. The results demonstrated that intervention sites, each having an assigned group including local stakeholders supported by a facilitator, lowered the neonatal mortality rate by 50% during the last intervention year compared with control sites. This process evaluation was conducted to identify and describe mechanisms of the NeoKIP intervention based on experiences of facilitators and intervention group members.

**Methods:**

Four focus group discussions (FGDs) were conducted with all facilitators at different occasions and 12 FGDs with 6 intervention groups at 2 occasions. Fifteen FGDs were audio recorded, transcribed verbatim, translated into English, and analysed using thematic analysis.

**Results:**

Four themes and 17 sub-themes emerged from the 3 FGDs with facilitators, and 5 themes and 18 sub-themes were identified from the 12 FGDs with the intervention groups mirroring the process of, and the barriers to, the intervention. Facilitators and intervention group members concurred that having groups representing various organisations was beneficial. Facilitators were considered important in assembling the groups. The facilitators functioned best if coming from the same geographical area as the groups and if they were able to come to terms with the chair of the groups. However, the facilitators’ lack of health knowledge was regarded as a deficit for assisting the groups’ assignments. FGD participants experienced the NeoKIP intervention to have impact on the knowledge and behaviour of both intervention group members and the general public, however, they found that the intervention was a slow and time-consuming process. Perceived facilitation barriers were lack of money, inadequate support, and the function of the intervention groups.

**Conclusions:**

This qualitative process evaluation contributes to explain the improved neonatal survival and why this occurred after a latent period in the NeoKIP project. The used knowledge translation intervention, where facilitators supported multi-stakeholder coalitions with the mandate to impact upon attitudes and behaviour in the communes, has low costs and potential for being scaled-up within existing healthcare systems.

## Background

In 2005, when the World Health Organization assembled experts around knowledge translation in global health, a main message was: *“Bridging the know–do gap is one of the most important challenges for public health in this century. It also poses the greatest opportunity for strengthening health systems and ultimately achieving equity in global health”*[1] p1. This statement reflects the dilemma that available knowledge, to a large extent, is not used in practice, despite the fact that it has the potential to improve healthcare services [2]. One such area where knowledge translation could make substantial improvements is the care of the newborn child. Annually, 3.3 million neonates die worldwide [3] despite the existence of simple, cost-effective and evidence-based interventions that could avert many of these deaths [4].

The Canadian Institute of Health Research defines knowledge translation as *“a dynamic and iterative process that includes synthesis, dissemination, exchange and ethically sound application of knowledge to improve health, provide more effective health services and products and strengthen the health care system”*[5]*.* There are many different strategies for translating knowledge into practice and some appear more effective than others [6]; for example, it has been demonstrated that methods involving social interaction, such as small-group meetings and multi-professional collaborations, are promising [7].

Facilitation has been described as a method with great potential [8-13]. This is a technique where one person (the facilitator) targets individuals or groups to make things easier by helping them to change their attitudes, habits, skills and ways of working [14]. Rather than presenting a simple linear concept where knowledge is transferred from an expert to a group in need of change, the facilitation method acknowledge that knowledge translation is complex and multifaceted [8-11,15]. During the last decade, facilitation has been evaluated as a method for improving health and survival among newborns in some low and middle income countries in South Asia, with positive outcomes on health indicators and survival [16-18]. For example, in the Makwanpur district in Nepal facilitators supported women’s groups to identify and formulate actions to address perinatal problems [17]. By this strategy neonatal mortality was lowered by 30% and coverage of antenatal care, institutional deliveries, skilled birth attendance and hygienic care increased. Although facilitation seems propitious, there is a shortage of projects using this approach as an intervention and thus, little evaluation of the process of implementation and its effectiveness exists [9,10,12].

Mortality among children under-five and neonatal mortality in Vietnam have declined faster than other countries [3,19-21]. However, considering that the Vietnamese government has focused on improving neonatal health [22], for example by launching national guidelines in reproductive health [23], the reduction of neonatal mortality has been slower than anticipated [24]. For three years (2008 – 2011) the *Neonatal health - Knowledge into Practice* (NeoKIP, Current Controlled Trials ISRCTN44599712) trial investigated the effectiveness of facilitation as a knowledge translation intervention for improved neonatal health and survival in the Quang Ninh province, Vietnam [25]. Laywomen recruited and trained to act as facilitators supported local commune groups involving primary health care staff and key persons engaged in perinatal health. These groups were assigned to identify and take action on local problems related to newborn health. The facilitators primarily used the quality improvement method ‘Plan-Do-Study-Act’ to help the groups to work in a structured way: i.e., identifying and prioritising local problems and appropriate actions (Plan), implementing the identified actions (Do), evaluating the results of the implemented actions (Study), and finally reconsidering the problems and performed actions (Act) [26]. During the 3 intervention years the groups identified 32 unique problems [27]. The most frequently identified problems dealt with antenatal care attendance, nutrition and rest during pregnancy, home deliveries, breastfeeding and postnatal home visits. To approach the problems, 39 unique actions were taken that mainly concerned communication to groups and individuals. In total more than 1,500 meetings took place with intervention groups and facilitators. During the first two intervention years there was no difference between the 44 intervention and 46 control communes regarding neonatal survival, but, for the third and last year the neonatal mortality rate (NMR) was 50% lower in the intervention communes [27]. Thus, having a multi-stakeholder group supported by a facilitator seemed to be a favourable intervention influencing the prevailing perinatal health situation in a commune. That NeoKIP was a complex social intervention requiring constant interpretation and negotiation among the involved stakeholders might explain the latent period of two years [27]. However, what worked and what did not work in the collaboration between the various stakeholders in this intervention deserved further exploration. Therefore, the present process evaluation aimed to identify and describe mechanisms of the NeoKIP facilitation intervention based on the experiences of facilitators and intervention group members.

## Methods

### The NeoKIP intervention

The NeoKIP trial was conducted as a randomised controlled trial in eight districts (90 communes) in Quang Ninh, a northern province of Vietnam [25]. By randomization, 44 communes were allocated as intervention communes and 46 as control communes. A commune in Vietnam is an administrative and political entity with 1,000 to 18,000 inhabitants. In each intervention commune, a multi-stakeholder group, called a maternal and newborn health group (MNHG), was constituted. Each group consisted of eight members; the vice-chairperson of the commune (having responsibility for health in the commune), three primary health care staff (typically a doctor, midwife and a registered nurse), a village health worker, two representatives from the Women’s Union (commune and village level) and a population motivator (providing counselling regarding family planning) from the National Committee for Population, Family and Children. In each MNHG, a chair was appointed (in most groups the vice-chairperson of the commune) to be responsible for the group’s activities between meetings and also during the meetings in collaboration with the facilitator. The purpose of having a mix of representatives with varying backgrounds in the MNHG was to understand and reach out at different levels in society and at the same time have the mandate to implement change. Each MNHG met monthly with a facilitator to identify local problems regarding neonatal health and to find and implement actions addressing these problems. The facilitators in the project were recruited from the Women’s Union organisation and trained for two weeks in group dynamics, quality improvement methods and basic evidence-based neonatal care, to be able to facilitate the work of the 44 MNHGs. Over the 3 intervention years, 11 women were employed for the 8 facilitator positions. The overall intention was that the facilitators should encourage and stimulate the intervention groups, being the possessors of both local and practical knowledge, to implement change adapted to the local situation of neonatal health and survival. Two researchers supervised and supported the facilitators, both in the field and through monthly gatherings throughout the intervention period.

### Design and study setting

This study is based on the interpretation of qualitative data using an emergent design [28], implying an expansion of the data collection throughout the intervention process.

The 8 districts where the NeoKIP study took place have approximately 350,000 citizens, a majority belong to the Kinh ethnic group and about 50,000 to any of the 10 existing minority groups. Health care is available at hospitals at regional, provincial and district level, and in each commune there is a commune health centre (CHC) providing primary health care [29]. The reproductive health care at the CHCs includes antenatal, delivery and post-natal care. However, CHCs situated in cities mainly provide counselling and comparatively simple procedures if operating in the same area as a hospital [30]. Village health workers are connected to each CHC, one based in each village, providing preventive care. Women and children can receive care at any of the levels in the healthcare system, however, hospitals charge patients with a higher user fee than the CHCs [31]. In the study area there were also private alternatives for antenatal care, but none providing delivery care. Besides the healthcare system, other organisations also serve the population regarding health care matters; the National Committee for Population, Family and Children provides family planning counselling and the Women’s Union supports women, particularly those belonging to poor and vulnerable groups.

Prior to the NeoKIP intervention, NMR was found to be 16 deaths per 1,000 live births (16/1,000) in Quang Ninh province, ranging from 10 to 44/1,000 in the different districts [32]. Primarily, newborns of mothers belonging to an ethnic minority group and living far away from a health facility had an increased risk of dying [33,34]. Further, home delivery was strongly correlated to NMR [29]. Data collection and analysis experiences of the facilitation intervention were collected through focus group discussions (FGDs) with facilitators and a selection of the MNHGs. Four FGDs (F1-F4) were performed with the facilitators at different time points (at start, and at 6, 27 and 36 month into the intervention). In the two FGDs conducted early in the intervention process, we used a moderator (LE) and a translator/note taker (DMD) who were well known to the facilitators and familiar with the NeoKIP project. For the last two FGDs we used an experienced moderator (VPNT) unknown to the facilitators in order to provide a context where facilitators more freely could express themselves. To capture the experiences of MNHG members, six out of the 44 MNHGs were purposely sampled [28] as study groups. The sampling was based on a variation of groups in terms of geography, facilitators acting in the groups and performance as a MNHG. The MNHG performance was estimated collectively by the facilitators and the researcher based on experiences from meetings with the groups. Thus, the six MNHGs were sampled from different districts (Table 1) and each of the MNHGs was enrolled in two rounds of FGDs (MNHG1 and MNHG2), 21 and 36 months into the intervention.

**Table 1:** Classification and characteristics of maternal and newborn health groups (MNHGs) selected for focus group discussions (FGDs)

**MNHG**	**Type of district**	**Participants in FGD**	**Age (mean)**	**Women (%)**	**Participants belonging to an ethnic minority group (%)**
1	Rural	8	45	88	0
2	Rural/Mountainous	9	43	56	22
3*	Rural	8/11	37	78/82	0
4*	Urban	8/9	42/40	78/86	0
5	Urban	8	41	88	0
6	Rural/Mountainous	8	41	75	100

*The MNHGs had different compositions at the two FGD rounds.

For the FGDs with facilitators, interview guides were designed with open-ended questions (and probes) suitable for the different time-points of the intervention period. For the FGDs with MNHGs, there was a similar interview guide for the two rounds containing open-ended questions (and probes) about experiences of the intervention and the facilitators work. The interview guides are presented in Additional file 1. The FGDs lasted from 60 to 120 minutes. All FGDs were audio recorded except the second FGD with the facilitators where the recording device did not work, and thus that FGD was excluded from analysis. For the remaining 15 FGDs the audio recordings were transcribed verbatim and translated into English to allow non-Vietnamese speaking researchers to participate in the analysis.

The analysis began with all 15 FGDs being read chronologically, to provide a naïve understanding of the data. This guided us to analyse the material in two separate data sets: the 3 FGDs with the facilitators and the 12 FGDs with the MNHGs. For each of the data sets, thematic analysis was applied based on the six phases described by Braun and Clarke [35]. First, the whole text was read through several times to obtain an understanding of the material and to search for meanings and patterns (phase 1). Then, the material was coded (phase 2), followed by a search for themes (phase 3). The initial themes were then closely reviewed individually and in relation to the entire data set and revised if necessary (phase 4). During the last two phases the themes were named (phase 5) and the results were written up by using a mix of text and extracts from the data sets to demonstrate each theme (phase 6). The first author (LE) conducted all steps of the analysis supported by ACE and LW. All authors were involved in verifying the codes, sub-themes and themes, which strengthened the credibility.

### Ethical considerations

The Scientific Committee at the Ministry of Health in Vietnam and the Research Ethics Committee at Uppsala University, Sweden, approved the study. All participants were informed about the purpose of the study and gave their verbal consent to participate.

## Results

The data analysis resulted in 4 themes and 17 sub-themes for the 3 FGDs with facilitators and 5 themes and 18 sub-themes for the 12 FGDs with MNHGs (Table 2). The results from each theme are presented by describing the content of the sub-themes together with illustrating quotes.

**Table 2:** Themes and sub-themes from focus group discussions (FGDs) with facilitators and maternal and newborn health groups (MNHGs)

		**Themes and sub-themes from FGD with facilitators**	**Themes and sub-themes from FGDs with MNHGs**
**Barriers**	**Money**	**Lack of money – a challenge for implementing a project successfully**	**A rare project without money**
		• Reimbursing all a necessity	• Salaries are needed, especially for low paid group members
		• Money a part of project culture in	
		• A lack of resources to include all	• Funds are necessary for running a project
		• Managing without money	
	**Support / Obstacles MNHGs**	**Support – an imperfect necessity**	
		• Facilitator training did not fit recipients needs	
		• Facilitators are supported by supervision if appropriately provided	
		• Lack of proper top-down support reduces the good spirit	
			**Obstacles for MNHGs to fully function**
			• Barriers to reach population and for population to reach health care
			• NeoKIP unknown to people
			• The MNHG did not function fully
			• MNHG members lacking knowledge
			• Organisations’ support needed for MNHGs
**Process**	**The facilitators**	**Being the facilitator is challenging, complex and requires versatility**	**The Facilitator – a new yet aporetic role**
		• Performance and skills increase over time	• The facilitator involves in meetings and activities in an enthusiastic way
		• Being a successful facilitator requires various skills and commitment	• The facilitator should be local and not change frequently
		• Lacking medical knowledge – an aggravating factor	• The facilitator - a person with surprising lack of clinical knowledge
		• Lacking confidence in the ability to function as facilitator	• The facilitator, an unnecessary person that neither provides nor receives support of importance
		• Adapting to local culture is key	
		• A good relationship between facilitator and MNHG, particularly the chair, facilitates a project	
	**The MNHGs**	**Facilitating a diverse group with conservative and hierarchical characteristics**	**Meet regularly, identify problems and choose communication strategies**
		• Joining several organisations in collaboration	• Regular meetings involving all, with chair as a decision maker
		• Facing negative attitudes and actions	• Targeting pregnant women first then newborns
		• Chairs’ behaviour influence group behaviour	• Communication – a universal solution for most targeted problems
		• Engagement and enthusiasm increased over time	
	**Impact**		**Nothing new and time-consuming, yet positive outcomes**
			• Nothing provided but words
			• Not new, but time-consuming
			• Increasing focus, knowledge and skills through collaborative group
			• Increased awareness and use of health care among population

### Experiences of facilitators

#### Being the facilitator is challenging, complex and requires versatility

The facilitators experienced that having a good relationship with the MNHG members, particularly the chair of the group, was key for working successfully within the group. These relationships were established by visiting the communes before the start of the intervention and by maintaining them throughout the intervention. To have a good relationship was considered important both for the function of the MNHG and for the confidence of the facilitator. Thus, when the chair of the MNHG was unwilling to collaborate, it became difficult for the facilitator to engage with the groups.

Facilitators described that being an experienced woman (regarding life and work) with social competence and good communication skills were important characteristics to allow them to manage the facilitator role. Further, a facilitator was required to be enthusiastic and engaging, while at the same time possessing the courage to confront MNHG members about their contributions, such as commitment during the MNHG meetings and activities in-between meetings. The facilitators expressed a lack of confidence in being able to manage this role, particularly at the beginning of the intervention. Further, the facilitators’ lack of clinical knowledge was an aggravating factor throughout the intervention and they described feeling inferior to the MNHG members and limited in their capacity. To mobilise MNHG members to attend the meetings and to write diary episodes after each meeting were necessary and time-consuming duties for the facilitators. To stimulate MNHG members at the meetings was challenging, especially if group members’ engagement declined. However, over time the facilitators’ overall skills improved and their satisfaction with acting as facilitators increased.

“I found it very hard to support the teams. This is because I don’t have professional knowledge while the other 8 members of a team have their own specialty, enough confidence and education … However, over time we received more knowledge and now at the end of the project, I find that my supporting role to the group is better, I can raise problems for discussion and I feel more confident about it.” (F4)

Fundamental to function well as a facilitator was to understand and adapt to the local context. Coming from the same geographical area as the MNHG made this easier for the facilitators; if they did not, it was necessary to learn about the local context in order to succeed within the MNHG.

#### Facilitating a diverse group with conservative and hierarchical characteristics

Combining representatives of several organisations together to collaborate and target a specific goal was considered a new and challenging yet appreciated way of working. The challenge with a multi-stakeholder group consisted of group members also having their own agenda in their respective organisations, while the appreciation of the group was the collaborative work towards a joint goal. The shared focus on a mutual goal also increased the dialogue between group members and their organisations.

The facilitators faced a lot of negative attitudes and actions, particularly in the beginning of the intervention. However, over time the engagement and enthusiasm among the MNHG members increased and gradually the groups understood their mission and enjoyed trying to improve the situation in their communes. The facilitators experienced the chair of the MNHG as an influential person whose behaviour affected other group members’ behaviour. With an unfocused chair, group members tended to become unfocused, while an interested and enthusiastic chair made group members engaged in the work for improved health in the commune. In some cases the MNHGs functioned well despite an absent chair.

“The group did very well although the chair of the group only joined once in a meeting. He did not know anything; he just sat there and kept silent during the meeting. When I invited him to join in the other meetings, he said: No I will not join; delivery is just the issue of women so the women should focus on that.” (F3)

Some facilitators described being successful where the MNHGs were established in a commune with a strong healthcare system, but failing in those with a weak one.

#### Support – an imperfect necessity

The facilitators experienced that the training they received in order to act as facilitators was insufficient. They thought that the training was too abstract and complicated and not adjusted to their level of knowledge.

“…there’s no need to use advanced words and to give examples from far away. They should use examples close to us. Training should be in the way that makes everyone understand. It should give detailed examples, such as how to act when I come to a meeting in a commune.” (F4)

The facilitators claimed that training to turn Women’s Union workers into facilitators should last over one month instead of two weeks. There were mixed perceptions about the support from supervisors; some appreciated their presence at the MNHG meetings and felt supported by this, while other facilitators believed that supervisors’ behaviour in the field inhibited the MNHG members and obstructed the collaboration between facilitators and the group. To function well in their new and difficult role, the facilitators thought it would have been beneficial to meet as a group, with or without a supervisor, more often than once a month. Overall, facilitators considered the support from the project and other organisations involved to be weak, which also impacted negatively upon the spirit among MNHG members.

#### Lack of money – a challenge for implementing a project successfully

There was a lack of understanding how a wealthy project (as NeoKIP was believed to be) could be implemented without financially supporting the MNHGs. NeoKIP was experienced as strange, in comparison to how other projects acted, for not supporting the MNHG members in their engagement. Besides not providing money for arranging the monthly MNHG meetings, it was also articulated that financial support was a necessity for implementing activities in the communes, for example to attract the public to come to a meeting.

“My recommendation is to give money to people participating in the propaganda meetings. They expect to get money. If we go there without money, it will be very difficult for them to listen. The custom in this area is like this.” (F4)

Further, without financial incentives, facilitators experienced that MNHGs could not implement actions in the most remote areas. As a consequence, the MNHGs were restricted to intervening in geographically close areas even though these were in less need. When the facilitators were criticised by MNHG members for the lack of financial support, facilitators explained the rationale for not funding meetings and activities, a process that was time-consuming but most often successful. More concerning for the facilitators was the two MNHG members (the village health worker and the head of Women’s Union from village level) having a very low salary from their organisations; facilitators believed they should be entitled to a basic salary from NeoKIP to assure that all in the group were reimbursed properly. These circumstances restricted the facilitators in creating a dynamic and positive group climate.

### Experiences of MNHG members

#### Meet regularly, identify problems and choose communication strategy

FGD participants described that the MNHG meetings were conducted on a regular basis with similarities in structure. The work in the MNHG required cooperation between people with different backgrounds from different organisations. All group members took part in the processes of identifying and implementing problems/actions, yet the chair of the group was often the decision maker. Primarily, the MNHGs focused on women’s health, sometimes in particular subgroups, such as adolescents, first time pregnant, unmarried, or those belonging to an ethnic minority group. The health of neonates was also targeted, but received less attention, particularly in the beginning of the intervention. However, neonatal death cases in the communes were often used as a starting point when identifying problems. Communication in multiple ways was the universal solution for all problems, being based on what is known as ‘communication papers’ (containing either clinically specific or more general information), most often produced by the midwife at the CHC. The papers were meant to be used in visits with commune members at their homes, at the CHC or when gathering in meetings. Various methods of communicating health messages were used, including the loudspeaker systems that exist in most villages which were used on a regular basis for airing messages.

“We have a tea shop at home so women sometimes come to see us in the evening for a cup of tea or to buy a noodle packet, so I provide information to any women I meet at home, and I also make home visits to their houses.” (MNHG1)

Most MNHG members were involved in the communication activities. However, some of them were perceived as more likely to have an impact on the public, for example, the chair of the MNHG. Further, when MNHG members met individuals, for example a young woman first time pregnant, they often chose to meet the whole family because it was believed to have greater impact than talking to the woman alone. MNHG members described that the way in which they communicated changed during the intervention.

“With the help of the NeoKIP project, I have changed my way of communicating. Before the project, I communicated without bothering about the result, without knowing whether the others heard what I said or not. Now, when I communicate I focus on changing them and making them hear me.” (MNHG2)

### The facilitator – a new yet aporetic role

The facilitator was mostly considered as an enthusiastic and friendly person that was helpful in improving the work in the MNHG. In general, the facilitators were apprehended by the MNHGs to perform poorly in the beginning of the project, however, over time they were perceived to have improved and were finally regarded as a valuable resource for the group. When meeting monthly, the facilitators helped the MNHGs to review and summarise their work, to discuss priorities and overcome difficulties. Although the facilitator was mainly seen as a person who helped the MNHG to meet regularly and focus, she was also participating in making decisions. It was appreciated when the facilitators shared experiences from other MNHGs. Besides facilitating monthly MNHG meetings, the facilitators also joined outreach activities when MNHGs implemented actions. As the facilitator was seen as a person with good communication skills, she sometimes also participated in communication activities arranged by the MNHGs.

Although MNHG members shared positive experiences of the facilitators, the critical voices about the facilitator role were loud; the facilitator was considered a person with surprising lack of clinical knowledge. While MNHG members had expected a facilitator with good clinical knowledge, they described that the facilitator’s lack of clinical experience slowed down the project. Thus, the intervention period was seen as a missed opportunity where the MNHG members could have gained a lot of knowledge if they had been facilitated by a person with superior knowledge.

“Actually, when there is a facilitator, support should be provided in terms of all aspects, including knowledge. But the facilitator of our group could not support us a lot. When she came and joined our monthly meeting, we summarised what we had done and planned for the next month. That’s it; there was no support on knowledge.” (MNHG1)

Further, it was experienced as novel but undesirable to be part of a project group where the members were supposed to gain knowledge by themselves. Therefore, if the facilitator role should be permanently established in the future, the MNHGs expressed a necessity of having a facilitator with clinical knowledge. Others described that the current facilitators had received poor training and support for their role, for example, some facilitators were not good at communicating, despite this being an integral part of their role. Study participants commonly became quiet when asked about the facilitators, a silence interpreted as either unvoiced criticism or a lack of understanding of the facilitator role.

### Nothing new and time-consuming, yet positive outcomes

MNHG members thought that the NeoKIP project targeted important patient groups but they were hesitant about accepting the facilitation method. Some described that facilitation only stimulated MNHGs to communicate messages to the public based on group members’ already existing knowledge. Others claimed that nothing was provided through the facilitation intervention, probably due to the fact that material, medicines or written documents were normally expected by the commune when a new project was implemented. As MNHG members experienced that nothing new was coming from the NeoKIP project, they occasionally prioritised other activities. Some members believed that the MNHG could meet less often, while others even thought that the project could stop.

However, the MNHG members also described several positive changes, both for their groups and the public; with NeoKIP the communes did the same as before but more, for example, more propaganda was reaching the remote areas with more detailed communication. The existence of the MNHGs also improved the relationships between the organisations represented in the groups, both at individual and organisational level. Furthermore, NeoKIP was considered positive because important stakeholders, together with the healthcare system, focused the MNHG participants’ efforts on reproductive health care issues. The joint work also increased the group members’ knowledge and skills in counseling and therefore their capacity. MNHG members believed that the quality of care had increased, perhaps because pregnant women were identified earlier than before and that women and children increasingly visited the CHCs for antenatal care, immunisation and varying kinds of consultancies.

“In my opinion, people’s awareness is increasing. They come to consult with us when they are 1–2 months pregnant, it means that they believe in us and want to talk with us…” (MNHG1)

In general, FGD participants had observed a reduction of home deliveries. Thus, the deliveries at the CHCs increased because the public thought of the CHC as a safer option than giving birth at home. Even the ethnic minorities were slowly changing their traditional ideas and increasingly utilised the care offered at the CHCs, such as being assisted during deliveries. The MNHG members perceived these changes as signs of an increased awareness among the public.

### A rare project without money

The MNHG members were unhappy about the lack of reimbursements for participating in a MNHG. They thought funding was a key element to motivate group members. Therefore, the lack of funding, particularly for salaries, was an obstacle for the effective functioning of the MNHGs. Most problematic in terms of there not being financial imbursement was the situation where the two group members had much lower levels of financial support from their organisations than the others (the village health worker and the head of Women’s Union from village level).

“I (village health worker) don’t have a salary from NeoKIP, only an allowance of 20,000 VND (1USD) per month for transportation. Truthfully, I joined the project as a social activity. When my children asked me if I have a salary when working with the project, I said ‘Nothing but smiles’.” (MNHG2)

MNHG members suggested that their work in the groups went beyond normal activities and therefore an additional payment was justified. However, they did continue to participate without a salary despite being unhappy about it. The lack of money further inhibited the MNHG members to identify and implement actions freely. FGD participants expressed that money was necessary to get good results. Money was needed when implementing actions, especially in the most remote areas, as transportation was costly. Furthermore, in the villages it was necessary to have money when interacting with the public, for example, when attracting them to meetings or during home visits.

### Obstacles for MNHGs to fully function

Although most of the MNHG members participating in the FGDs planned to maintain the MNHG structure and activities after the intervention, they did experience disturbances which prevented the MNHGs from functioning fully. The public first became aware of the MNHGs and their activities if being exposed to any of their actions. According to MNHG members, this was unfortunate and was due partly because of the lack of ceremonies when NeoKIP was launched. It was also suggested that the MNHG meeting should be integrated with already existing meetings at the CHC, as the MNHG would be strengthened with more participants from village level. MNHG members further described that their lack of knowledge, both clinically and regarding communication methods, contributed to a lower quality of care. To increase knowledge, more training and support by superior clinicians, such as a paediatrician, was suggested. To get support from the MNHG members’ organisations was also recognised as a necessity to function fully.

The FGD participants mentioned a number of barriers to their reaching the population and for the people to reach the CHC. Long distances and poor roads were key barriers, which mainly had consequences for those in most need of help, for example, people living in mountainous regions or on boats who often were poor and/or belonged to an ethnic minority group. Further, interfering relatives was another barrier for care seeking, and poverty was a barrier that could hinder a woman to exclusively breastfeed her child as she needed to work. In addition, it was considered time-consuming to implement actions, especially in villages where several languages were spoken. However, people in general were considered helpful in these areas and assisted as translators.

“There was a case where the pregnant woman wanted to give birth at the CHC, but the relatives, because of limited understanding, wanted her to stay home. In that case she stayed home because her parents and siblings did not bring her to the CHC and she could not go herself.” (MNHG1)

## Discussion

In this qualitative process evaluation of a facilitation intervention, both facilitators and MNHG members concurred that having intervention groups consisting of representatives of various organisations was beneficial for targeting newborn health. Further, the facilitators were perceived to have an important role in assembling these groups every month. In all FGDs, participants also expressed some impact of the NeoKIP intervention, such as increased knowledge and increased care seeking behaviour among the public, as well as enhanced quality of care and capacity of staff. However, achieving these improvements was a slow and time-consuming process. Barriers for the intervention process to function smoothly were evident, for example, facilitators experienced barriers related to their support while MNHG members articulated barriers regarding their ability to achieve changes in the communes because of the function of the MNHGs. In fact, this process evaluation reveals a number of factors that potentially influenced the stakeholders involved and thus, the NeoKIP trial [27]. Three primary influencing factors were money, the facilitator role and viewing the MNHG as a coalition; factors which need consideration if scaling up this type of intervention in the Vietnamese context.

The objective of the NeoKIP trial was to evaluate facilitation as a knowledge translation strategy [25]. To do this, NeoKIP was set up as a randomised controlled trial where intervention sites differed from control sites by having a multi-stakeholder group receiving monthly support by a facilitator. MNHG members did not receive additional pay for participating in the NeoKIP trial as group activities were supposed to be integrated into the group members’ ordinary assignments. Further, to reimburse MNHG members would have reduced the opportunities to evaluate the effectiveness of the trial. It would also have increased the costs of the trial, and thus reduced the possibility of scaling-up the intervention. However, the lack of money was articulated as a problem in the FGDs, that is to say money was suggested to be necessary for arranging monthly MNHG meetings and for implementing actions and for supporting group members. A study assessing the effects of the economic reforms on the Vietnamese healthcare system during the 1980s identified that the salaries within the healthcare sector declined, which resulted in loss of trained staff, poorer service and lowered morale of healthcare staff [31]. Consequently, healthcare staff needed additional incomes through other pursuits to be able to support themselves. In a more recent study in northern Vietnam, Dieleman and colleagues [36] report that primary health care staff still consider their salaries from the healthcare system to be insufficient for daily life and having a second job to be a necessity, a coping strategy also identified in neighbouring countries to Vietnam [37]. Hence, the critical voices regarding lack of money in the NeoKIP intervention was likely a reflection of MNHG members’ dissatisfaction regarding their salaries and work situation. Bearing this in mind, it was surprising that facilitators could convince MNHG members that additional money from the NeoKIP project was not the solution for having actions implemented in a sustainable manner. In addition, the adequate critique about the low paid group members in the MNHGs highlights a dilemma in Vietnam, where, for example, village health workers, who are part of the healthcare system, are expected to perform basic health care in their communes on a voluntary basis [36]. Yet, we believe that the focus on lack of money has consumed much of the facilitators’ and MNHG members’ energy and time, which could have been allocated to their mission to improve newborn health. The project should have put more effort into making the reasons for not financially supporting the MNHGs clear from the outset and gained prior approval from involved stakeholders in order to reduce the discussions of money throughout the project.

The facilitators’ experiences of their role in this study are in line with previous descriptions suggesting the facilitator to have a multifaceted role [10] involved in a multifaceted process [9]. In the current study the facilitators experienced that they needed to possess various characteristics and skills to become successful, including; social competence, communication skills, enthusiasm, and courage to challenge group members. Similar qualifications have also been identified in previous studies [8-11,13]. Further, in a study where facilitators were involved in interprofessional education, weekly gatherings were recognised as being an important component for the facilitators’ progress [38], suggesting that the development of skills of the NeoKIP facilitators might have been faster with more frequent support.

As the MNHG members expected to receive knowledge from the NeoKIP project, they highlighted that the most obvious deficit with NeoKIP was the facilitators’ lack of health knowledge. However, our findings suggest that the facilitators also lacked skills in facilitating groups, such as managing group dynamics and communicating effectively. These deficits, most apparent in the beginning of the intervention, might have influenced the MNHG members to see the facilitators as unnecessary and incompetent individuals. For some MNHG members, the facilitators’ lack of health knowledge was a barrier throughout the entire intervention, while others identified the facilitator as a resource for the MNHGs despite being a layperson in relation to healthcare. Overall, the facilitators’ skills, both regarding health knowledge and of facilitation, were perceived to improve over time, which might contribute to explain why the positive results of the NeoKIP intervention did not occur until the last year of the project [27]. The NeoKIP facilitators were trained to use a helping and enabling approach rather than telling and persuading MNHG members what to do, in line with Harvey and colleagues’ recommendations regarding the facilitator role [10]. However, the NeoKIP facilitators sometimes made decisions for the MNHGs and influenced their selection of problems and actions, indicating a deviation from these principles. The persuading and exhorting behaviour of the facilitators might have been an attempt to meet MNHG members’ expectations of them as facilitators. This finding suggests that a change agent who possesses superior knowledge compared with her/his target groups might have worked better in the Vietnamese context, such as an opinion leader or through educational outreach [12]. An opinion leader is described to be a knowledgeable, influential and trustworthy person working internally in an organisation and engaged in supporting change [12,39,40], while educational outreach visits are performed by a person external to an organisation having more knowledge than the practitioners that he/she supports [10,40]. However, while such individuals were not available in the study area, this is an important issue considering the need for a cost effective intervention that is feasible to scale up. Thus, for the current trial we believe that recruiting individuals from the Women’s Union and training them to become facilitators was adequate. However, the results indicate a need to provide facilitators with more healthcare knowledge than we did in NeoKIP in order to sufficiently familiarise them with MNHGs’ focus areas and more thoroughly support their skills in facilitating groups.

The NeoKIP intervention, which was a bottom-up approach using local multi-stakeholder groups, succeeded to lower neonatal mortality substantially [27]. The function of the MNHG was in line with the description of a community coalition, which is a group of people who agree to work together to achieve a common goal, such as to introduce a solution to health problems by using existing resources [41]. In the theory of coalition, Gamson [42] suggests that four parameters can predict if a coalition can succeed: *The initial distribution of resources (1)* indicating what resources the members bring to a coalition, and *the payoff for each coalition (2)* indicating the future benefits from their actions. Further, *the non-utilitarian strategy preferences (3),* that is to say the inclination among coalition members to join with each other regardless of their control of resources, and *the effective decision point (4),* which signifies the crucial type and amount of resources required to control a decision. O’Neill et al. [43] suggest the theory of coalition to be useful when understanding intersectoral health interventions, yet suggest the inclusion of a fifth parameter to the theory: *organisational context (5).* Organisational context refers to the rules set by the environment, relating to decisions such as number of players in the coalition and whether to have or not to have a meeting. In this study, the MNHG was formed in order to involve several organisations from various levels in society [25]. According to the theory of coalition, the MNHG members had a smorgasbord of resources to establish a coalition. Further, as all MNHG members represented organisations that in one way or another were stakeholders in perinatal health, they had an inclination to participate in this coalition as it benefited their organisations. However, several barriers emerged that might have reduced these preferences: lack of money, MNHG members having duties outside the MNHG, the work in a MNHG being regarded as time-consuming and that the facilitators lacked health knowledge. Further, some FGD participants expressed that group members jointly took decisions while others claimed that the chair of the group was the main decision-maker. There might be power structures within the MNHG that we have not detected relating to the fifth parameter (*organisational context*). For example, in the Vietnamese healthcare system, decisions and communication are often directed from the top to the bottom rather than through a dialogue [44]. Therefore, the facilitators with a background from the Women’s Union could have disturbed the coalition when having the role of facilitating the group while originally possessing a lower rank then several of the MNHG members. However, the fact that some facilitators were accepted by MNHG members indicates that they succeeded to assume their facilitator roles and support the NeoKIP intervention.

### Limitations

Thematic analysis was chosen as the method for analysis to identify patterns and draw conclusions from experiences made by stakeholders involved in the NeoKIP intervention [45]. Descriptions of thematic analysis and how it actually has been used vary considerably [35,45,46]. In this study we explicitly chose to follow the steps described by Braun and Clarke [35]. However, we made two exceptions from their suggestions; first, in the result section we have not included a comparison between our findings and findings in the literature, and secondly, due to a large sample size, we have restricted the use of quotes. We believe that the large number of focus groups, including data from both facilitators’ and MNHG members’ experiences at several occasions over the intervention period, has strengthened the analysis and thus the findings of the mechanisms of the facilitation process.

## Conclusions

Previous facilitation interventions in South Asia where facilitators have supported women’s groups have been successful in reducing neonatal mortality and improving care practices. However, it has not been known if a facilitation intervention implemented into the public sector system could be effective. The NeoKIP trial was a novel complex social intervention where facilitators supported local multi-stakeholder groups that succeeded to improve neonatal survival after a two year latent period. The findings in the current qualitative process evaluation indicate that the facilitators improved their skills over time and functioned best if they were familiar with the local culture and were able to come to terms with the chair of the group. Although the MNHGs did not function impeccable, to combine representatives from several organizations within a coalition was experienced as being a beneficial strategy providing mandate to change attitudes and behaviour in the commune. Two identified barriers for the current facilitation intervention were the facilitators’ lack of health knowledge and the absence of financial support for MNHG members. These barriers can contribute to explain why the impact of the facilitation intervention was delayed, in terms of a reduction in NMR. However, the choice of facilitators and to not reimburse MNHGs were deliberate strategies which presumably contributed to making this facilitation intervention successful after a latent period and suitable for being scaled up within healthcare systems.

## Competing interests

The authors declare that they have no competing interests.

## Authors’ contributions

LW, LE and TQH designed the intervention while DMD and TQH were responsible for training and supervision of facilitators. LE, DMD, TQH, MM and LW designed this study where VPNT and LE moderated the focus group discussions with assistance from DMD. VPNT, DMD and TQH assured that translations were correct. LE was responsible for data analysis and drafted the manuscript, with assistance of ACE and LW. All authors have read and approved the final manuscript.

## Pre-publication history

The pre-publication history for this paper can be accessed here:

http://www.biomedcentral.com/1471-2393/13/234/prepub

## Supplementary Material

Additional file 1 Interview guides for Focus Group Discussions with facilitators and Maternal and Newborn Health Groups (MNHGs). Click here for file
