# Supplementary material for: Lessons learned from stakeholders in a facilitation intervention targeting neonatal health in Quang Ninh province, Vietnam
Source: BMC Pregnancy Childbirth. 2013 Dec 13;13:234. doi: 10.1186/1471-2393-13-234 (PMC3866580; doi:10.1186/1471-2393-13-234)
Supplement: Additional file 1 — Interview guides for Focus Group Discussions with facilitators and Maternal and Newborn Health Groups (MNHGs). [file 1471-2393-13-234-S1.doc]

# Additional file 1. Interview guides for Focus Group Discussions with facilitators and Maternal and Newborn Health Groups (MNHGs)

**Interview guide for the initial Focus Group Discussion with Facilitators (Questions in bold and probes within parenthesis)**

- **What is your reflection from the course in May?** (Did you feel well prepared for your task as a facilitator after the course? Could we have trained you in a different way?)
- **How has the preparations been before the first meeting/s, e.g. your meetings with the chairmen?** (Did you have a pre meeting with each chairman? What else have you been doing to prepare yourself for the start of the intervention?)
- **How was the first meeting/s with the MNHGs?**
- **How does the facilitation work for you?** (In your role as a facilitator, what is most difficult? – Communication, use of tools, rules for the group, record-keeping, etc. Do you get much focus in the group?)
- **How is your interaction with the group?** (How do you look upon the group-members and how are their attitudes to you? Are you respected as a person and as a facilitator?)
- **How is your interaction/collaboration with the vice-chairmen?**
- **How are the group members’ contributions in the groups?** (Are all contributing, some not at all and some too much; are the group members interested in these meetings and interested to get them to work?)
- **How is the collaboration with the supervisor/s and monthly meeting progressing?** (Are you having a close contact with supervisor/s, are the monthly meetings happening and are they useful for you? Can we change anything in the existing way of working?)
- **How is the documentation working for you and the group?** (Diaries, minutes and other documentation in-between meetings?)
- **To get the facilitation to develop and work better in the future what can we do?**

(Regarding you as facilitators and we as supervisors/researchers)

- **You have now finalised your first month as a facilitator, what is your general impression so far?** (Difficult, terrible, fun, challenging, etc.)

**Interview guide for the follow-up Focus Group Discussions with Facilitators**

**(Questions in bold and probes within parenthesis)**

- **What are your experiences from working as facilitators?** (Has the facilitator role been difficult to perform? Too difficult? Were you as active women union members suitable for this role? Have you received sufficient training and support to accomplish your role?)
- **What have you achieved during these years of working as a facilitator?** (Regarding MNHGs, your facilitation role and you personally?)
- **What have been the main barriers for making progress with the MNHGs?**
- **How do you experience the MNHG members?** (Have they been keen on working in NeoKIP? Have they been treating you with respect? Have they understood your role in the group? Has it been a fruitful collaboration with MNHG members?)
- **If the NeoKIP idea would be tested in another Vietnamese province, what changes would you suggest?** (To have persons with a different background trained as facilitators? Have a different setup of training, support and supervision? Meet groups more or less frequent? To have a different economic support for facilitators and/or MNHGs?)

**Interview guide for the initial Focus Group Discussions with MNHGs (Questions in bold and probes within parenthesis)**

- **What is your experience from working in a MNHG?** (What are your thoughts on working with maternal and neonatal health? Have your perceptions on this issue changed over time?)
- **Can you tell what your group specifically has done to improve maternal and newborn health care?** (Do you see any changes because of the work of your groups? If changes have happened, in what way: To you in your profession, to the care practices at the CHC, to the Women Union and/or to the overall situation in the commune?)
- **What is your opinion about the facilitator in your group? What do you think of the methods the facilitator has used?** (Has the facilitator been helpful in getting the group to work? To make changes of maternal and neonatal care? Has the role of the facilitator been clear? Have the methods the facilitator introduced been helpful?)
- **What have influenced your MNHG to achieve its objectives? What helped/enabled your group’s performance? What are the barriers/constraints of your work? (**Conditions/circumstances within the MNHG, within the health care system or outside the health care system?)
- **Have you found the work in the MNHG meaningful? What are your thoughts on a continuation of the work?** (Has the group been working in a fruitful and efficient way? Have you appreciated the work? Would you like this process to continue?)

**Interview guide for the follow-up Focus Group Discussions with MNHGs**

**(Questions in bold and probes within parenthesis)**

- **We met more than one year ago. How has the work in your MNHG proceeded?**
- **If you look at the whole intervention period, what are the most important problems that your group has tackled?** (What actions have your group implemented to improve maternal and newborn health care during these 3 years? Have these activities made an impact?)
- **Can you tell me if the work of your group has developed during these 3 years?**

(Are you working in the same way as in the beginning or has the work of the group developed during these years? Has it been a raise or decline in how active your group has been?)

- **In the Focus Group Discussions conducted more than one year ago, several of the members in the six MNHGs expressed that NeoKIP is not a new way of working. Has NeoKIP been beneficial in improving healthcare practice?** (Is there a potential for this way of working in the future? What changes could be made to adapt to this way of working?)
- **What are your experiences from working with a facilitator?** (Has the facilitator been helpful in getting the group to work in your meetings? Has the role of the facilitator been clear? Has the facilitator been helpful in accomplishing change of perinatal practice? Has the type of facilitator that NeoKIP has offered been useful? Has the skills and the performance of the facilitator developed over the three years of the project?)
